# Beyond accreditation: Specialist training as a strategic national asset in South Africa

**DOI:** 10.4102/jcmsa.v4i1.478

**Published:** 2026-07-24

**Authors:** Francis E. Smit, Johannes J. Fagan, Zach M. Koto

**Affiliations:** 1 College of Cardiothoracic Surgeons, The Colleges of Medicine of South Africa, Cape Town, South Africa; 2 The Colleges of Medicine of South Africa, Cape Town, South Africa

## Introduction

South Africa’s specialist training system is one of the country’s most valuable yet underappreciated national assets. It underpins highly specialised healthcare delivery and ensures health system sustainability through workforce development, clinical leadership, research and innovation. Producing specialist physicians and surgeons requires a complex partnership involving universities, provincial health departments, academic hospitals, the Health Professions Council of South Africa (HPCSA), the Colleges of Medicine of South Africa (CMSA) and national government.

Postgraduate medical education occurs entirely within clinical environments. Registrars acquire skills while simultaneously delivering vital patient care. Consequently, quality of training is inseparable from the functionality of the underlying health system. Deficiencies in staffing, infrastructure, equipment, governance or clinical capacity compromise both patient care and educational outcomes.

While accreditation traditionally assured educational quality, its outcomes increasingly offer insight into the health of the healthcare system itself. Accreditation findings therefore should extend beyond compliance and should serve as indicators of institutional resilience, governance effectiveness and future workforce capacity.

## Specialist training and national interest

Developing a medical specialist requires 12–15 years of sustained public investment, supported by sophisticated clinical infrastructure. The return on this investment extends far beyond the individual. Specialists serve as clinical leaders, educators, researchers and innovators whose influence shapes healthcare performance for decades.

The constitutional foundations of specialist training are clear. Section 27 of the South African Constitution guarantees access to healthcare services,^1^ while Section 29 affirms the right to further education.^1^ Specialist training contributes directly to both obligations. However, no single institution can independently fulfil this responsibility. Universities provide academic leadership; provincial health departments maintain clinical platforms; the HPCSA establishes accreditation standards; and the CMSA assesses knowledge and competence. Success depends upon effectively aligning these institutions. When tensions arise between service delivery, fiscal constraints, educational requirements and regulatory expectations, the consequences affect the entire system.

This interdependence is most evident within academic health complexes and joint academic complexes, which operationalise cooperative governance. These structures represent the nexus where service delivery, education, research and workforce development converge.

When fiscal pressures, leadership instability, infrastructure failures or staffing shortages affect these environments, the consequences extend beyond immediate service disruptions. Registrar training opportunities diminish, research output declines and future specialist capacity is compromised.

## Accreditation as a mechanism for patient protection

At its core, accreditation exists to protect patients at three interconnected levels (See Table 1). Firstly, it safeguards current patients by ensuring appropriate supervision and safe clinical practice. Secondly, it protects registrars by ensuring access to the clinical exposure, mentorship and educational resources necessary for professional development. Thirdly, it protects future patients by ensuring graduates possess the competence required for independent specialist practice.

**TABLE 1:** Accreditation as an early-warning system for healthcare.

Domain	Immediate consequences	Long-term consequences
Patient care	Reduced specialist services	Diminished access to specialised healthcare
Education	Reduced supervision and clinical exposure	Lower specialist production
Workforce	Recruitment challenges and filling of vacancies	National specialist shortages
Governance	Accreditation concerns and programme instability	Institutional decline and reduced system resilience

Importantly, accreditation findings indicate governance risk. The erosion of specialist training programmes rarely occurs abruptly. Instead, it emerges through accumulating vulnerabilities: unfilled trainer and registrar posts, declining procedural volumes, deteriorating infrastructure, equipment failures and weakening leadership. Accreditation reviews identify these vulnerabilities long before they manifest as service collapse or significant workforce shortages. Viewed through this lens, accreditation functions as an early-warning system identifying threats to educational quality, service sustainability and future workforce supply.

## Towards shared national stewardship

South Africa requires a shift from a compliance-driven approach towards collective stewardship and shared accountability. We propose a *National Compact for Specialist Training and Academic Health Services*. This compact would provide a collaborative framework through which the National Department of Health, provincial departments, universities, academic health complexes, the HPCSA, registrar representatives, the private sector and the CMSA can jointly safeguard training capacity. This would not require additional bureaucratic structures; rather, it would strengthen coordination, transparency and accountability across existing institutions.

A National Compact would focus on monitoring integrated system performance indicators, including specialist trainer and registrar vacancy rates, accreditation outcomes, examination performance, infrastructure functionality and service capacity. These measures provide an early warning framework to identify risks before they threaten programme sustainability. Reframing accreditation transforms it from a regulatory endpoint into a strategic instrument for workforce planning, risk mitigation and health system strengthening.

South Africa is renowned for its robust medical and dental training. However, current challenges threaten to reverse these gains.^2^ Against this background, a national imbizo is necessary to co-create a sustainable, uniquely South African training paradigm. Preserving specialist training capacity is a constitutional obligation, a workforce imperative and a national strategic priority. Ultimately, the resilience of South Africa’s healthcare system depends on how successfully it protects and sustains the institutions making such training possible. Specialist training must be managed as a strategic national asset whose stewardship is shared to shape the health of future generations.
